# Interruption of enteral nutrition in the intensive care unit: a single-center survey

**DOI:** 10.1186/s40560-017-0245-9

**Published:** 2017-08-04

**Authors:** Midori Uozumi, Masamitsu Sanui, Tetsuya Komuro, Yusuke Iizuka, Tadashi Kamio, Hiroshi Koyama, Hideyuki Mouri, Tomoyuki Masuyama, Kazuyuki Ono, Alan Kawarai Lefor

**Affiliations:** 1 Emergency and Critical Care Medicine, Dokkyo Medical University, Mibumachi, Shimotsuga-gun, Tochigi, Japan; 2 Department of Anesthesiology and Critical Care Medicine, Division of Critical Care Medicine, Jichi Medical University Saitama Medical Center, 1-847 Amanumacho, Omiya-ku, Saitama-shi, Saitama, 330-8503 Japan; 3 Department of Surgery, Jichi Medical University, 3311-1 Yakushiji, Shimotsuke-shi, Tochigi 329-0498 Japan

**Keywords:** Interruption of enteral nutrition, Diagnostic procedures, Therapeutic interventions, Nutritional protocol, Energy deficit

## Abstract

**Background:**

Interruption of enteral nutrition (EN) in the intensive care unit (ICU) occurs frequently for various reasons including feeding intolerance and the conduct of diagnostic and therapeutic procedures. However, few studies have investigated the details of EN interruption practices including reasons for and duration of interruptions. There is no standard protocol to minimize EN interruptions.

**Methods:**

This is a retrospective review of 100 patients in the ICU staying more than 72 h and receiving EN in a 12-bed, medical/surgical ICU in a tertiary care center in 2013. Data collected include total time designated for EN; the number of EN interruption episodes; reason for each interruption categorized as diagnostic study, therapeutic intervention, or gastrointestinal (GI) event, and their individual subcategories; duration of each interruption; and the presence of written orders for interruptions.

**Results:**

One hundred patients staying in the ICU for at least 72 h and receiving EN were included. There were 567 episodes of EN interruption over a median ICU length of stay of 17.1 (interquartile range 8.0–22.0) days. There were a median of three EN interruption episodes per patient. EN interruption was performed for undetermined reasons (166 episodes, 29%), airway manipulation (103 episodes, 18%), GI events (78 episodes, 14%), and intermittent dialysis (71 episodes, 13%). Median duration of EN interruption in all patients was 5.5 (3.0–10.0) h. The cumulative interruption time corresponds to 19% of the total time designated for EN. Duration of EN interruption varied according to reason, including airway manipulation (9.0 [5.0–21.0] h), tracheostomy (9.5 [7.5–14.0] h), and GI events (6.5 [3.0–14.0] h). The average calorie deficits due to interruptions were 11.5% of daily target calories. Only 60 episodes (12%) had clear written orders for interruption.

**Conclusions:**

Based on this single-center retrospective chart review, interruption of EN in the ICU is frequent, reasons for and duration of interruption varied, and airway procedures are associated with a relatively longer duration of interruption. Documentation and orders were frequently missing. These results warrant development of a protocol for EN interruption.

## Background

In the intensive care unit (ICU), the interruption of enteral nutrition (EN) occurs frequently for various reasons including feeding intolerance, and the conduct of diagnostic and therapeutic procedures [1–3]. However, guidelines for administration of EN to critically ill patients [4–6] only indicate that “efforts should be taken to reduce EN interruptions due to diagnostic and therapeutic procedures” [5, 7], without offering specific protocols for minimizing EN interruptions [6].

In fact, there are several existing studies on the effect of EN interruption [2, 3, 8], suggesting that EN interruption is common in the ICU. However, one study did not evaluate the interruptions associated with diagnostic procedures [3], and another study failed to assess the duration of the interruptions [8]. No studies have apparently investigated the effectiveness of EN interruption protocols in the literature.

To establish effective interruption protocols in the future, obtaining accurate information regarding the current status of EN interruptions is an important initial step. Therefore, we conducted a single-center retrospective observational study to evaluate the frequency, duration, and reasons for EN interruptions, and the presence of written orders for interruption.

## Methods

This study was approved by the Institutional Research Ethics Review Committee, and consent for research participation was waived due to the retrospective study design. The study was performed in a 12-bed combined surgical and medical ICU at the Jichi Medical University Saitama Medical Center, Saitama, Japan. Patients aged 18 years or older who stayed in the ICU for 72 h or longer and who commenced EN from January 1 to December 31, 2013, were included. Data collection was terminated when a study patient was discharged from the ICU or placed on either an oral diet or intermittent EN. All data were retrospectively retrieved from hospital electronic medical records (COSMOS®, IBM, Tokyo, Japan) and the electronic ICU chart system (PIMS®, Phillips, Tokyo, Japan).

As an institutional practice at the time of study initiation, the initiation of EN within 48 h of ICU admission was encouraged unless contraindicated [5]. In patients for whom EN was indicated, incremental continuous feeding was initiated at 20 kcal/h for the energy and protein target, calculated as 25 kcal multiplied by ideal body weight and 1.2–1.5 g/ideal body weight. Every morning, a daily goal for energy and protein administration was determined at the physician’s discretion and documented in the electronic chart. If a patient was considered stable enough to tolerate a full diet, the rate of continuous feeding was increased by 20 kcal/h every 4 h. Measurement of gastric residual volume was not routinely performed but was performed if events such as gastric discomfort or fullness, obvious regurgitation and active vomiting occurred. In those cases, metoclopramide and erythromycin were used to promote gastric motility with transient interruption of the EN at the discretion of the physician. However, definitive protocols regarding interruption of EN did not exist except that an effort was made to achieve the daily goal by increasing the rate of administration after restarting EN. Once a patient was stabilized with a daily nutritional target achieved by continuous administration of EN at a rate of 60–80 kcal/h, intermittent administration or daytime continuous administration (e.g., 7 am–7 pm) was allowed at the physician’s discretion.

Data collected include the total administration time of EN (from start to end), the number of EN interruption episodes, the proportion of daily target calorie administration missed due to interruptions, the reasons for EN interruption (e.g., diagnostic testing, therapeutic interventions, and gastrointestinal (GI) events), duration of each interruption, time interval from the beginning of the interruption to beginning of the procedure, time interval from the end of the procedure to resumption of EN, the presence or absence of physician orders for EN interruption and its resumption, and the presence or absence of an endotracheal tube.

Reasons for interruption were categorized as follows: “undetermined” for patients where a clear reason for interruption could not be identified; “GI event” for abdominal pain, significant gastric residual volume, vomiting, diarrhea, or gastrointestinal bleeding; “airway manipulation” for intubation, cricothyroidotomy, tracheotomy tube replacement, successful tracheal extubation, or extubation attempt for situations where weaning of the ventilator was attempted but patients did not pass the spontaneous breathing trial, and for cases where the clinical load did not allow the extubation of those patients; “T-piece trial” for liberation from the ventilator using a weaning protocol; “tracheostomy” for tracheostomies performed either in the ICU or the operating room; “ICU diagnostic and therapeutic procedures” for bronchoscopy, transesophageal echocardiography, or placement or removal of central venous lines, extracorporeal membrane oxygenation, or other devices and endoscopy; “Procedures outside of the ICU” for radiological diagnostic procedures or interventions performed outside of the ICU; “Intermittent dialysis” for intermittent dialysis performed in either a dialysis suite or ICU; and “daytime only administration” for cases where EN was administered during the daytime only for the purpose of preventing airway trouble during the night.

The proportion of calorie deficits in daily caloric goals due to the interruption (%) was calculated as daily calorie deficits due to the interruption divided by the daily caloric goal. Daily calorie deficits (kcal) due to interruptions were calculated by subtracting the actual energy administered from a daily caloric goal determined at the physician’s discretion, both of which were retrieved from the ICU electronic medical record.

For data presentation, categorical variables are expressed as numbers (%), and continuous variables are expressed as mean ± standard deviation or median with interquartile range (IQR), as appropriate.

## Results

Patient demographics are shown in Table 1. Between January 1 and December 31, 2013, a total of 100 patients stayed in the ICU for at least 72 h and received EN. Patient age was 66.7 ± 14.4 years, and 63 (63%) patients were men. Acute Physiology and Chronic Health Evaluation II score was 21.8 ± 6.8. A total of 95 (95%) patients were endotracheally intubated at the time of study inclusion, ICU stay was 17.1 (8.0–22.0) days, and in-hospital mortality was 19%. There were 567 episodes of EN interruptions, including 515 episodes in intubated patients (90%). The number of EN interruptions per patient was 3.0 (1.0–5.3) (Table 1).

**Table 1:** Baseline characteristics of study patients (*N* = 100)

Characteristic	Value
Age, mean (SD)	66.7 (14.4)
Male gender, *N* (%)	63 (63.0)
APACHE II score, mean (SD)	21.8 (6.8)
Intubated (endotracheal) patients at time of study inclusion, *N* (%)	95 (95.0)
ICU length of stay, median (IQR) (days)	17.1 (8.0–22.0)
Diagnosis, *N*	
Cardiovascular surgery	57
Surgical	6
Neurosurgical	2
Medical	35
Total episodes of EN interruption, *N*	567
Episodes in endotracheally intubated patients, *N* (%)	515 (90.8)
Number of EN interruptions per patient, median (IQR)	3.0 (1.0–5.3)
Caloric deficit of daily caloric goal due to EN interruptions, %	11.5
EN interruption orders documented, %	12.0
EN resumption orders documented, %	8.0
Number of patients with a feeding tube placed post-pyloric region	11
Number of patients given prokinetic agents	34

*APACHE II* Acute Physiology and Chronic Health Evaluation II, *SD* standard deviation, *IQR* interquartile range, *EN* Enteral nutrition, *ICU* Intensive Care Unit

**Fig. 1:**
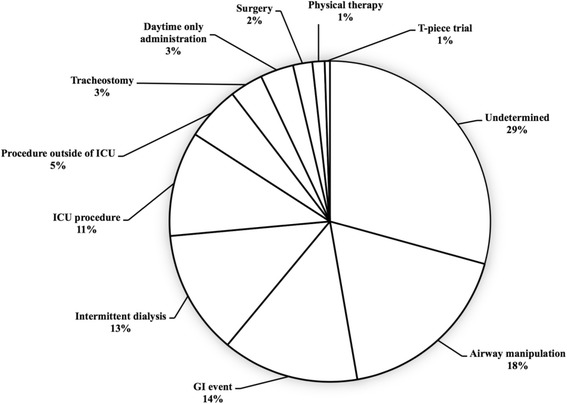
Reasons for EN interruption

The most common reason for EN interruption was undetermined (166 episodes, 29%), followed by airway manipulation (103 episodes, 18%), GI events (78 episodes, 14%), intermittent dialysis (71 episodes, 13%), and ICU diagnostic and therapeutic procedures (61 episodes, 11%) (Fig. 1).

Median values and IQR of duration of EN interruption by category are shown in Table 2. The median duration of EN interruption in all patients was 5.5 (3.0–10.0) h. The proportion of EN interruption duration to total EN administration was 19% (data not shown). Events with relatively long total EN interruption include surgery, airway manipulation, and daytime-only administration. Events with a large variation in interruption duration were GI events, airway manipulation, and undetermined. Events with a relatively short total EN interruption duration include ICU diagnostic and therapeutic procedures, T-piece trials, and physical therapy.

**Table 2:** Duration of enteral nutrition interruption per episode

Reason for enteral nutrition interruption^a^	Time, median (IQR), h
Total	5.5 (3.0–10.0)
Gastrointestinal event	6.5 (3.0–14.0)
Airway manipulation	9.0 (5.0–21.0)
T-piece trial	3.0 (2.5–4.5)
Tracheostomy	9.5 (7.5–14.0)
Surgery	29.6 (11.0–34.3)
Intermittent dialysis	6.0 (5.0–8.0)
Procedure outside of ICU	4.0 (3.0–6.0)
ICU procedure	4.0 (2.0–5.5)
Physical therapy	1.0 (1.0–2.5)
Daytime only administration	10.0 (9.0–10.5)
Undetermined	4.0 (2.5–6.0)

^a^Detailed explanation of each category are documented in the text

*IQR* interquartile range

The time intervals between EN interruption and procedure start are shown in Table 3. The time interval from EN interruption until the event (procedure, etc.) start was 1.2 (0.3–3.7) h. Relatively long intervals between EN interruption and procedure start were documented for tracheostomy (5.8 [4.0–9.3]). The time from the end of an event until EN restart was 1.8 (0.8–4.2) h (Table 4). In a majority of the reasons for EN interruption, a substantial delay for restart of EN was documented. After tracheostomy, 3.2 (1.0–3.6) h were consumed until EN restart. Also, not significantly long but substantial delays were detected for intermittent dialysis, procedures outside of the ICU, and ICU procedures. Relatively large variations were found for most reasons of EN interruption including intervals from EN interruption to procedure start and the interval from procedure end until EN restart.

**Table 3:** Interval between enteral nutrition interruption and start of procedure

Reasons for enteral nutrition interruption^a^	Time, median (IQR), hours
Total	1.2 (0.3–3.7)
Airway manipulation^b^	3.9 (1.8–5.8)
T-piece trial	2.3 (2.0–2.3)
Tracheostomy	5.8 (4.0–9.3)
Surgery	6.2 (0.5–9.8)
Intermittent dialysis	0.3 (0.0–0.6)
Procedure outside of ICU	0.6 (0.3–0.7)
ICU procedure	1.3 (0.5–2.5)
Physical therapy	0.0 (0.0–0.5)

^a^Detailed explanation of each category is documented in the text

^b^Patients in whom the ventilator weaning and tracheal extubation attempt failed, or patients where clinical load did not allow extubation were not included in this category

*IQR* interquartile range, *ICU* intensive care unit

**Table 4:** Interval from end of procedure to restarting enteral nutrition

Reasons for enteral nutrition interruption^a^	Time, median (IQR), h
Total	1.8 (0.8–4.2)
Airway manipulation^b^	7.7 (3.0–21.8)
T-piece trial	0.3 (0.2–0.3)
Tracheostomy	3.2 (1.0–3.6)
Surgery	7.0 (1.7–16.8)
Intermittent dialysis	1.0 (0.3–1.8)
Procedure outside of ICU	1.2 (0.5–2.3)
ICU procedure	1.5 (0.7–3.0)
Physical therapy	0.6 (0.3–2.0)

^a^Detailed explanation of each category are documented in the text

^b^Patients in whom ventilator weaning and tracheal extubation attempt failed, or patients where clinical load did not allow extubation were not included in this category

*IQR* interquartile range, *ICU* intensive care unit

Average calorie deficits in daily caloric goal due to interruptions were 11.5% (Table 1). EN interruption orders were clearly documented for only 12% of the total interruption episodes, and the EN resume orders were present in 8% (Table 1).

## Discussion

This retrospective observational study in a single-center mixed ICU demonstrates that EN interruption is frequent, relatively long, and associated with substantial calorie deficits for various reasons. Airway procedures are associated with relatively longer durations of interruption compared to other reasons. Documentation and orders are frequently missing.

Previous studies [2, 8, 9] have reported GI dysfunction and interruptions due to therapeutic procedures to be the most common reasons for EN interruption. Although the current study observed similar trends, previous studies did not categorize the procedure details [2, 8, 9], and invasive procedures were excluded [3]. To our knowledge, this is the first study to evaluate the relationship between the reasons for and duration of EN interruption.

To achieve the goal of minimizing calorie deficits while preventing EN-associated complications, nutritional protocols should focus on minimizing the interruption of nutrition along with enhancing administration. To establish an efficient interruption protocol, it is essential to evaluate the duration of the interruption for each procedure since different procedures have different safety margins for the duration of the interruption. Each interruption can be divided into several components. Whether the time of EN interruption before or after the procedure has a greater impact on nutritional deficits is a valid question since the cause for delay and the longest part of the interruption duration differs among procedures. McClave et al. investigated the durations and reasons for interruption of EN in a medical ICU [2], showing the proportion of interruption in the total duration of EN administration for an individual reason, but did not present the actual length of time for the interruption [2]. Passier et al. described the duration of interruptions, partitioning into two durations before and after the procedures, but the numbers presented in the study were only the average durations among all patients and were not individualized according to the reasons for interruption [3]. Adam et al. investigated interruptions and their reasons and energy deficits in five ICUs in the UK [8], but a thorough description of the interruption reasons was not performed in this study. In contrast with these studies, we describe the duration of the interruption for each reason, and the duration of interruptions for the procedure and from the time of the procedure to restart for each reason.

The duration of EN interruption related to either patient GI symptoms or respiratory and airway manipulations was long, and large variations found. Large variations were observed for interruptions for diagnostic tests or therapeutic interventions, both in the time from EN interruption until treatment start and the time from treatment end until EN restart. Reasons for the long, varying durations of these interruptions may be due to a lack of a clear interruption protocol, leading to a long unnecessary interruption, and a delay in early restart after a procedure. External factors including excess clinical load in the ICU and other departments involved in patient care may affect the timing of diagnostic and therapeutic procedures. Extended duration until EN restart after procedures may be due to insufficient staff awareness of the calorie deficit caused by the interruption, which might worsen patient outcomes [1]. Intervals between EN interruption and tracheostomy (5.8 [4.0–9.3] h) could be shortened in at least half of the patients, if the general consensus of a 6-h interruption before the procedure is followed. Substantial delay until restart of EN could also be improved for tracheostomy (3.2 [1.0–3.6] h), intermittent dialysis (1.0 [0.3–1.8] h), procedures outside of the ICU (1.2 [0.5–2.3] h), and ICU procedures (1.5 [0.7–3.0] h).

In this study, the average nutritional deficit due to EN interruption for the daily caloric goal was 11.5%. Adam et al. [8] reported an average deficit in prescribed calories of 24% in five facilities studied, while other studies have reported calorie deficits of 13–40% [9–11]. The results of the current study are better than those in previous reports, probably due to efforts to supplement substantial calorie deficits, not by reducing the duration of the interruption but by increasing the rate of administration after restarting EN. However, depending on an increased rate of administration is a potential source of GI complications of EN. In one ICU with an EN administration protocol, calorie administration was closer to prescribed targets compared to ICUs without a protocol [8]. Although details of this protocol are unknown, EN administration protocols may affect the resulting calorie deficit.

In the current study, the most common reason for EN interruption was “undetermined.” Clear orders for EN interruption and resumption were not documented in most cases. Insufficient nutrition in critically ill patients is related to increased mortality rates [1]. Many institutions adopt an efficient EN protocol for achieving calorie targets and reducing interruption by EN intolerance. However, a practical protocol for reducing interruption time due to the procedures has not yet been made available [12]. Busy ICU staff may fail to pay sufficient attention to nutritional deficits due to interruptions but would become more cautious of the interruption if a clear interruption protocol existed.

The current study has several limitations. First, a single-center design may hamper generalizability of the results. Nutritional policies and practices in an individual ICU vary and change over time. These findings may not be applicable in a substantial number of ICUs. However, it is noteworthy that even in a closed ICU as in the study institution, the absence of an interruption protocol and documentation may result in calorie deficits. These data may be of value in institutions considering implementation of a protocol, especially in open ICUs. Second, due to the retrospective study design, various errors could be possibly included. However, data from an electronic chart system, as in the current study, could allow more accurate documentation of the initiation and cessation of EN and amounts given than by a manual system.

## Conclusions

This retrospective single-center study evaluated details regarding interruption of enteral feeding administration and shows that durations of interruption were long for a variety of reasons. In addition, the most frequent reasons for interruption were undetermined, and documentation and orders were frequently missing. These findings may be of use in institutions considering the development and verification of an interruption minimizing protocol.

## Abbreviations

EN: Enteral nutrition
GI: Gastrointestinal
ICU: Intensive care unit
IQR: Interquartile range
